# CyberKnife Radiotherapy for Skull Base Petroclival Metastases Including Dorello’s Canal: Report of 10 Cases

**DOI:** 10.7759/cureus.10692

**Published:** 2020-09-28

**Authors:** Shinichiro Miyazaki, Yuko Harada, Yusuke Sasaki, Takanori Fukushima

**Affiliations:** 1 CyberKnife Center, Shin-Yurigaoka General Hospital, Kawasaki, JPN; 2 Neurosurgery, Tokyo General Hospital, Tokyo, JPN; 3 Neurosurgery, Duke University, Durham, USA

**Keywords:** skull base metastasis, abducens nerve palsy, cyberknife, dorello's canal

## Abstract

Skull base petroclival metastases cause diplopia due to abducens nerve palsy. Diplopia is visually disabling, and skull base metastasis is extremely difficult to treat even with microscopic surgery. However, stereotactic radiotherapy with CyberKnife (Accuray Incorporated, Sunnyvale, California) has been very successful in 10 cases. As the abducens nerve runs through Dorello’s canal in the skull base, the radiation dose and fraction were adjusted to avoid damage to the nerve. Since these metastases are not located inside the brain but in the skull base, contrast magnetic resonance imaging (MRI) combined with fluorodeoxyglucose-positron emission tomography (FDG-PET) was essential to detect the cancers.

## Introduction

Skull base metastasis causes pain or cranial nerve palsy, which are disturbing symptoms for patients. In 1981, Greenberg et al. classified the neurological findings of skull base metastases into five syndromes: orbital, parasellar, middle fossa, jugular foramen, and occipital condyle [1]. in 2018, Hayashi et al. reported metastatic tumors in the clivus or petrous, which presented diplopia due to abducens palsy. These tumors comprised 20% of skull base metastases [2] We treated 10 patients with diplopia who presented with petroclival metastases, which falls into the category of parasellar syndrome or middle fossa syndrome by Greenberg.

Since the surgical approach into the skull base is extremely difficult, the first choice of treatment is radiotherapy. The CyberKnife System (Accuray Incorporated, Sunnyvale, California) is a robotic radiosurgery system that provides highly precise stereotactic radiotherapy (SRT). SRT with CyberKnife is non-invasive, and hospitalization is not required.

We have successfully treated 10 cases with skull base petroclival metastases with CyberKnife SRT. Contrast MRI and FDG-PET revealed petroclival metastases, thus we suspected the cancer should be in Dorello’s canal through which the abducens nerve runs. The treatment required some adjustment to decrease the compression pressure to the abducens nerve in Dorello’s canal without damaging the nerve. This is the first report to treat diplopia by treating petroclival metastasis with multi-fractionated SRT, presuming that the tumor is compressing the abducens nerve in Dorello’s canal.

## Materials and methods

All the patients with diplopia underwent contrast-enhanced brain MRI and FDG-PET/CT scans to evaluate the entire brain and body prior to treatment. If the brain MRI revealed petroclival metastasis and FDG uptake was observed in the same area, it was suggested that the abducens nerve was compressed by the metastatic tumor in the petroclival area on the skull base. Standard surgery was difficult in all cases, as the tumors located in the skull base were difficult to reach manually.

Multisession stereotactic radiotherapy was performed for 10 cases using the CyberKnife G4 system. All cases presented metastatic cancer which caused abducens nerve palsy.

Tumors were tracked with the skull-tracking algorithm. The gross tumor volume (GTV) was defined as a visible tumor on enhanced MRI with images merged for target definition. GTV was considered the same as the clinical target volume (CTV). The planning target volume (PTV) included CTV and a margin of 1.2 mm.

In order to decompress the tumor (decrease the tumor volume in order to decrease pressure to the abducens nerve) in Dorello’s canal without damaging the abducens nerve, the radiation fractions were nearly doubled.

The treatments were on an outpatient basis and followed up every four to six months with MRI or FDG-PET.

## Results

Table 1 shows the results of 10 cases.

**Table 1:** Details of 10 cases Histology, location, age, sex, PTV of tumor, radiation dose, prescription isodose line, radiation fraction, follow-up period, and outcome of 10 cases No adverse events were observed. PTV: planning target volume

Case	1	2	3	4	5	6	7	8	9	10
Histology	pharyngeal adenoid cystic carcinoma	pharyngeal cancer	pharyngeal cancer	breast cancer	breast cancer	thyroid follicular cancer	lung adenocarcinoma	uterus cancer	prostate cancer	adenocystic carcinoma in paranasal sinus
Location	L	L	L	L	L	L	R	R	R	R
Age	56	53	54	46	47	45	66	67	59	42
Sex	M	F	M	F	F	M	M	F	M	F
PTV (cc)	24.3	9.89	55.58	11.8	13.5	3.9	0.37	7.4	4.8	6.58
Dose (cGy)	4500	4500	4800	3500	4200	3500	3250	3800	4000	3400
Fraction	15	8	10	8	7	5	5	5	5	5
Prescription isodose line (%)	72	72	68	67	76	80	90	76	82	72
Follow-up period	11 months	3 years 9 months	3 years 5 months	3 years 8 months	3 years 1 month	4 months	1 year 7 months	1 year	8 years 5 months	8 months
Outcome	Tumor disappeared, diplopia resolved	Tumor disappeared, diplopia resolved	Tumor disappeared, diplopia resolved	Tumor disappeared, diplopia resolved	Tumor disappeared, diplopia resolved	Tumor decreased, diplopia resolved	Tumor disappeared, diplopia resolved	Tumor disappeared, diplopia resolved	Tumor disappeared, diplopia resolved	Tumor disappeared, diplopia resolved

There were three cases of pharyngeal cancer, two cases of breast cancer, one of paranasal cancer, one of uterus cancer, 1 prostate cancer, 1 lung cancer, and 1 thyroid cancer. PTV ranged from 0.37 cc to 55.58 cc, and fractions ranged from 5 to 15. Radiation dose ranged from 3250 cGy to 4800 cGy. All patients recovered from diplopia.

**Figure 1:**
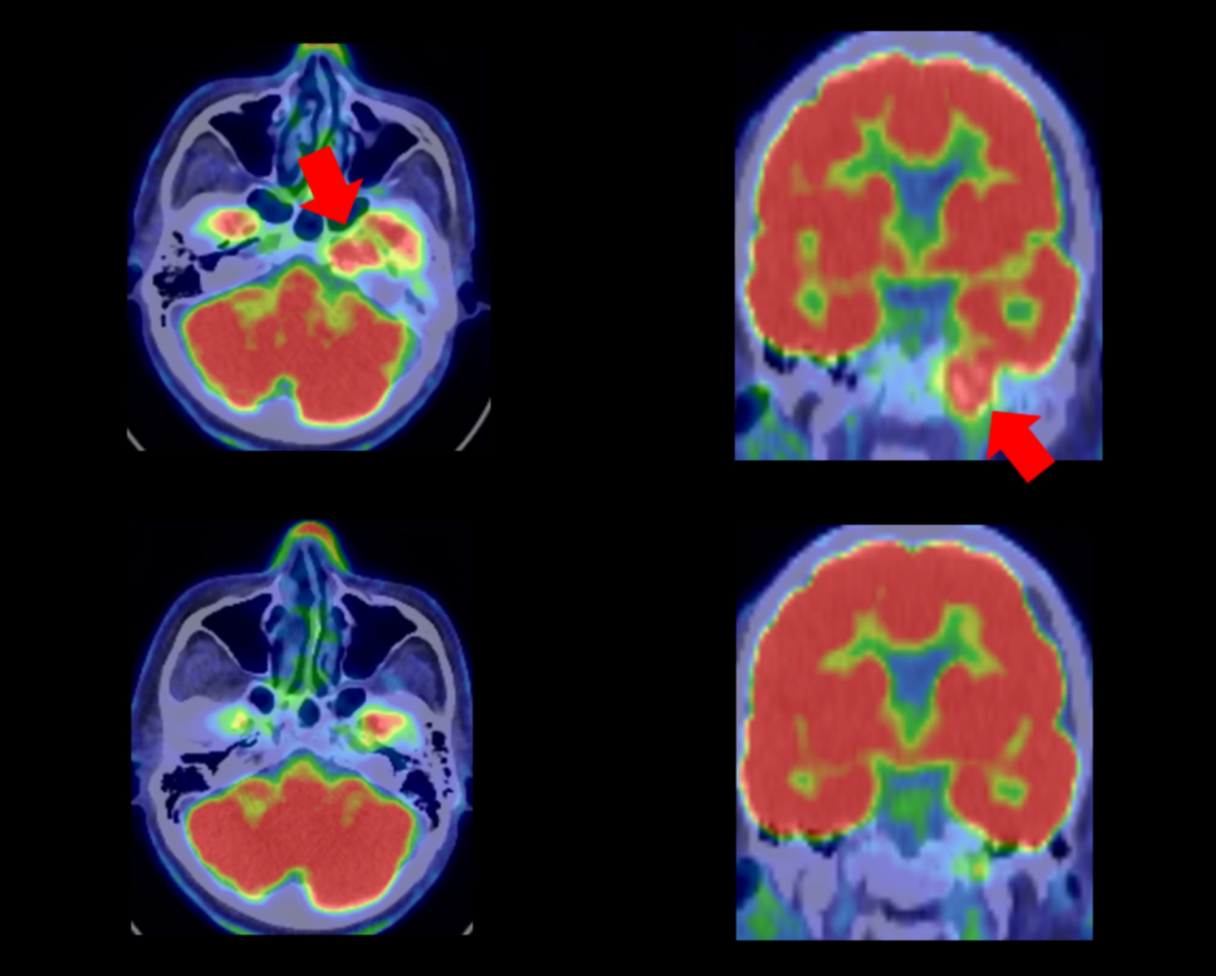
FDG-PET of Case 1 FDG-PET before CyberKnife (top) and after CyberKnife (bottom). The tumor (red arrow) disappeared after treatment. FDG-PET: fluorodeoxyglucose-positron emission tomography

The FDG-PETs of Case 1 before and after treatment are shown in Figure 1. Petroclival metastasis was remarkably decreased in only six months.

**Figure 2:**
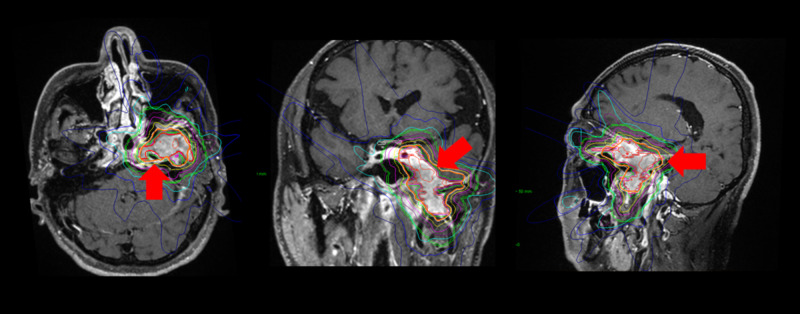
CyberKnife treatment plan for Case 1 Contour maps were drawn around the tumor (red arrow).

The Case 1 treatment plan of CyberKnife radiation therapy is shown in Figure 2. The contour map with isodose lines is shown drawn around the tumor. 

**Figure 3:**
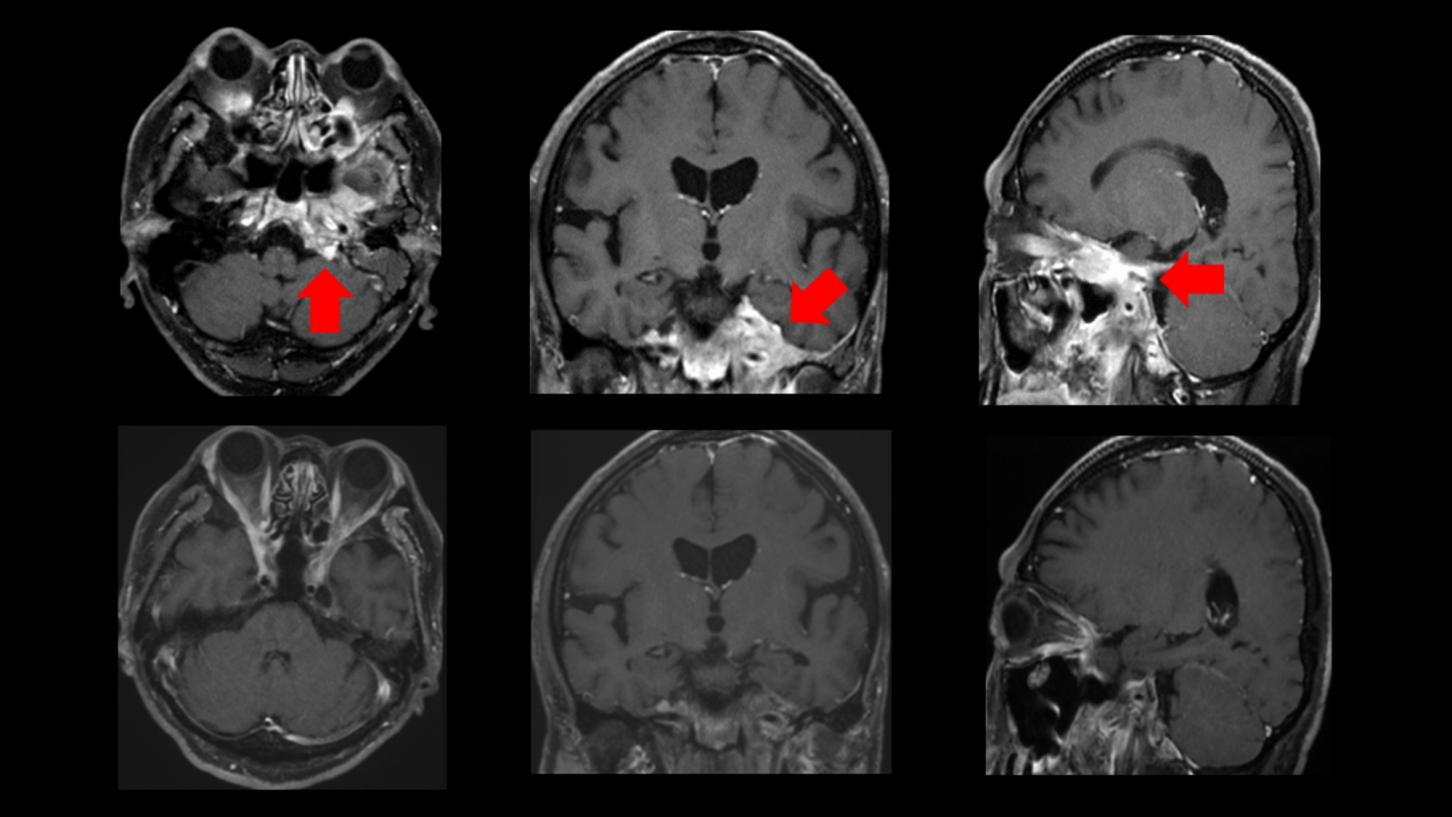
Contrast-enhanced MRI of Case 1 Contrast-enhanced MRI before CyberKnife (top) and after CyberKnife (bottom). The tumor in the petroclivus (red arrow) disappeared after treatment. MRI: magnetic resonance imaging

Contrast-enhanced MRI of Case 1 is shown in Figure 3. Petroclival metastasis was easily detected by FDG-PET rather than MRI; however, MRI is more suitable for making the CyberKnife treatment plan.

**Figure 4:**
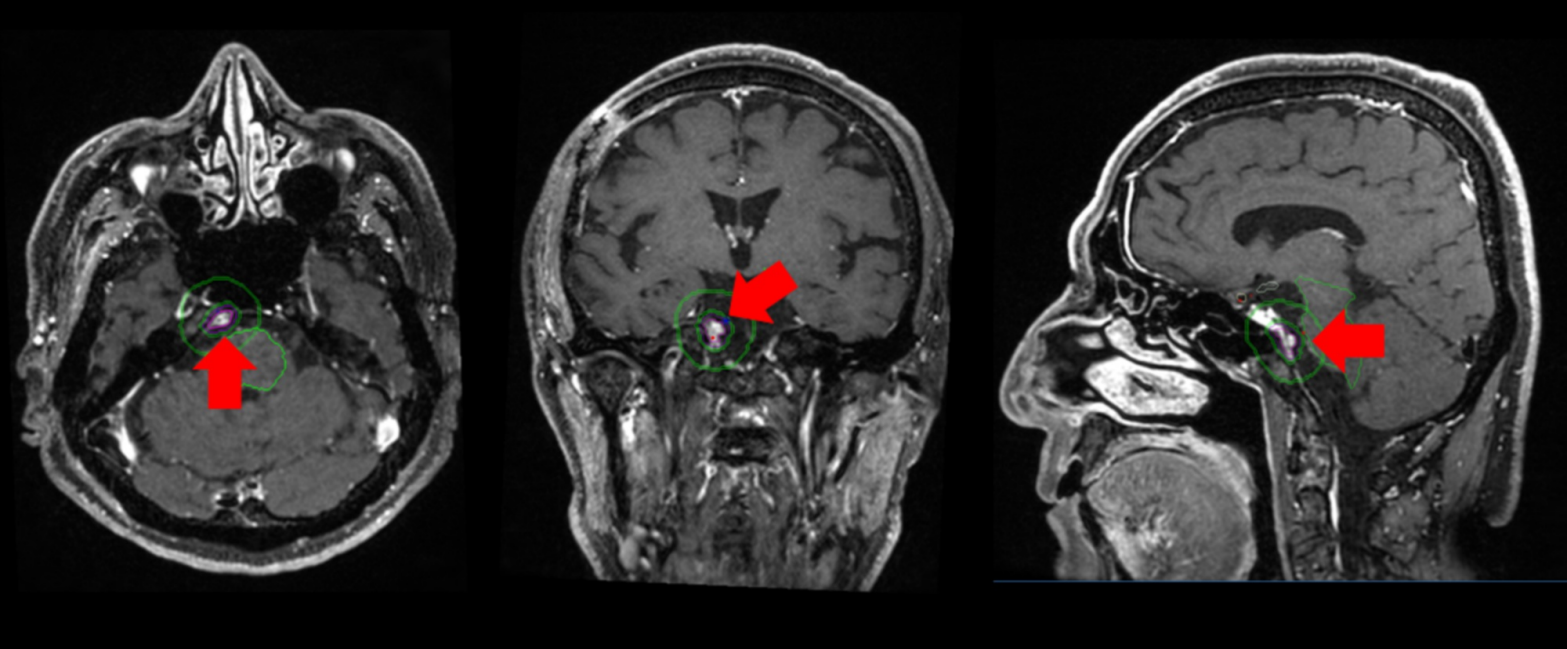
CyberKnife treatment plan of Case 7 Contour maps were drawn around the tumor (red arrow).

Case 7 presented with diplopia after the therapy of lung cancer. Contrast-enhanced MRI revealed a small tumor in the petroclival area. The treatment plan of CyberKnife radiation therapy is shown in Figure 4.

**Figure 5:**
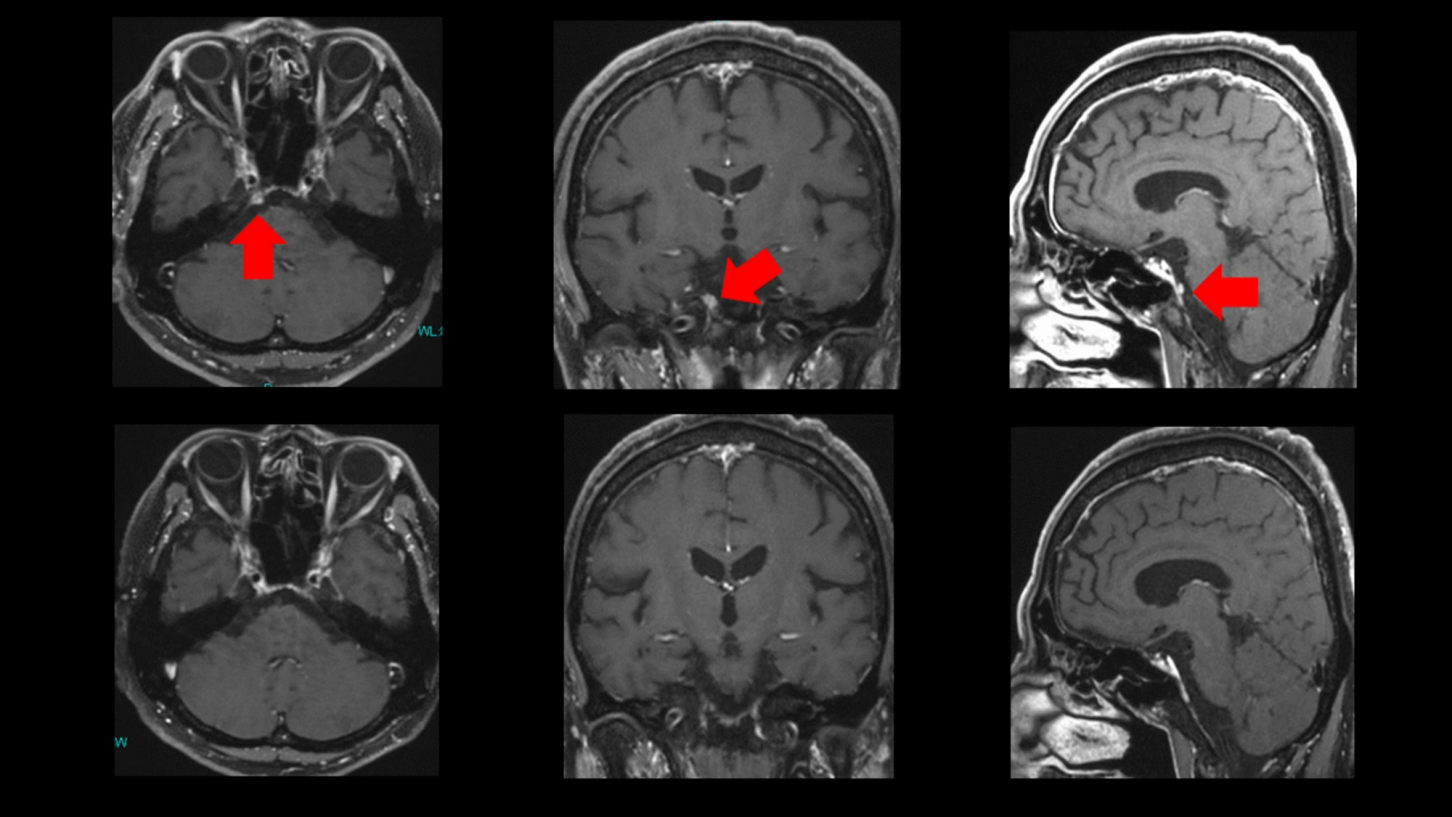
Contrast-enhanced MRI of Case 7 Contrast-enhanced MRI before CyberKnife (top) and after CyberKnife (bottom). The tumor located in Dorello's canal (red arrow) disappeared after treatment. MRI: magnetic resonance imaging

The tumor disappeared in six months as shown in Figure 5. Diplopia disappeared in only three months.

## Discussion

Dorello’s canal is an osteofibrous passage formed by a narrow depression near the tip of the petrous bone, located behind Gruber’s petrosphenoidal ligament, between the petrous apex and the clivus [3]. The abducens nerve passes through this canal. Dorello identified the enclosed region as a potential site of abducens nerve constriction, leading to abducent palsies [3-4]. Dorello’s canal is widely recognized as a key landmark in skull base surgery of the petroclival region and holds clinical significance due to its relation to the abducens nerve and surrounding vascular structures [3,5].

The abducens nerve is fully exposed from the posterior fossa through its entrance into Dorello’s canal [6]. After entering Dorello’s foramen, measuring 6 to 12 mm in length and 1 to 3 mm in width, the nerve is covered by a sleeve of dura propria for a variable distance (several millimeters) towards the cavernous carotid artery [6,7]. If cancer migrates into this canal, it will compress the abducens nerve to cause diplopia. It is, therefore, extremely difficult to remove the cancer from Dorello’s canal even with microsurgery. Conventional radiation therapy typically requires 30-day treatment, whereas SRT with CyberKnife requires only several days. SRT also provides high precision in three dimensions. Furthermore, conventional radiation risks damaging surrounding tissues and organs, which are precluded with SRT. SRT is, therefore, clearly optimal with some special adjustment to critically avoid any damage to the abducens nerve in the canal.

There are several reviews about radiation therapy on skull base tumors. Chamoun and DeMonte wrote in their review that radiotherapy is the main form of treatment for skull base metastasis, and the general recommendation for radiotherapy is 35 Gy in 14 fractions over three weeks [8]. Clump et al. reported the efficacy of SRT, with the most common fractionated regimen being 24 Gy delivered in three fractions, which improved symptoms within one month [9]. Mori et al. reported that if skull base metastasis and skull base invasion are relatively localized, they can be good candidates for SRT [10]. However, optimal treatment protocols or guidelines have not yet been established. Due to the relative rarity of skull base metastases and complicated structures such as Dorello’s canal, a radiation regimen needs to be composed for each patient [8].

Contrast MRI and FDG-PET have made it possible to detect small lesions of skull base metastases. The brain normally uptakes FDG while the non-cancerous skull does not. If the skull uptakes FDG, metastasis is demonstrated. FDG-PET also simultaneously reveals the location of primary cancer in the body. Therefore, FDG-PET is highly critical in detecting skull base metastasis and its primary cancer.

This study clearly demonstrates the successful treatment of diplopia by CyberKnife radiotherapy for skull base petroclival metastases. The keys of success were utilizing contrast MRI and FDG-PET for treatment planning and the hyper-fractionated method to avoid any damage to the abducens nerve compressed by tumors in Dorello’s canal.

## Conclusions

Ten cases of diplopia due to petroclival metastases were successfully treated with CyberKnife SRT. The challenging treatment of these tumors was successful due to the precise detection of tumor locations by contrast MRI and FDG-PET and CyberKnife SRT.
